# Torture of the Dying

**Published:** 1897-01-23

**Authors:** 


### TORTURE OF THE DYING.

Is it merely a silly prejudice that makes us look with solemn awe on the hour of death, and makes us feel that however troublous a man's life may have been his last moments should be shielded from bodily suffering and protected from mental torment?

We think not; and we believe that all will agree that when a man's work is done, when the hour of dissolution is at hand, when that complex thing called life is on the point of ceasing, such consciousness as remains to him should at least be left in peace.

The law, however, does not think so, and every resident in every hospital must have witnessed the torture of the dying, the miserable restoration to consciousness of brains which are being mercifully clouded by approaching death, which is involved in the taking of a dying deposition.

If a man's life had been passed in the promotion of some great aim, or the pursuit of some prolonged investigation, and death threatened to carry away the yet unwritten product of all his work, and to deprive his ambition of its reward, there would be some excuse; or if the criminal guilty of great offences were offered the chance of one final explanation, that he might confess his soul and make peace with men, there might again be some excuse. But it is not so. The harmless and unwilling witness of a crime, the innocent sufferer from an unprovoked assault, or the participator in wrong-doing long since atoned for, may be roused up from his dying lethargy and made to tell the hateful tale and enter afresh into details, all of which he had hoped were long buried in the past. Conscience, truly, doth make cowards of us all, but that conscience should be made a whip to scourge us with, and to make us cowards in our dying hours, is too bad, especially when one considers the objects for which the whip is so heavily laid on. Perchance it may be to help justice in its pursuit of crime, but quite as probably may it be that some wretched divorce may go more smoothly, or that some filthy libel may be more easily defended. To realise the cruelty of the process, it must be remembered that two things are necessary for the validity of a dying deposition. The dying man must still be conscious and know what he is about; but, on the other hand, he must be so ill that all hope of recovery shall have left his mind. In a case a little while ago a woman entirely invalidated her depositions by adding the words "But I intend to try" to her statement that she knew she could not get better. If everyone died happily, if everyone passed away from this life in the firm confidence of a joyful resurrection, this would not matter. But we know it is otherwise. The large majority drift into unconsciousness with but small thought of the future; but if they think, then the terror of uncertainty comes upon them. And here comes the cruelty. The very essence of the dying deposition is that they shall think; the one thing on which its validity depends is that the sufferer shall be fully conscious that for him there is no hope, and that, whatever the thought of death may rouse in his enfeebled mind, death certainly is before him. Nor is this allowed to be a vague impression; it must be positively expressed in cruel words. Those who have seen the process with all its uncertainties; have watched the tendency of the patient to drift in and out of consciousness, to wander back to old memories, and to babble on irrelevant but happier themes; who have witnessed the repeated necessity of bringing back the patient to the point, and forcing him to think of what he would so willingly forget; and who all the time have seen how plastic the failing brain is in the hands of the questioner, must agree with the remarks made a few days ago by Mr. Justice Hawkins in reference to a statement made by a dying woman. He is reported to have said that he had read it with a great deal of pain, and he thought it a great pity that the last hour of the poor creature's life should have been spent in a cross-examination of this kind. This is both kind ness and common sense. When "Swift to its close ebbs out life's little day" let us at least have peace.

